# Characterization of the Neuroinflammatory Response to Thiol-ene Shape Memory Polymer Coated Intracortical Microelectrodes

**DOI:** 10.3390/mi9100486

**Published:** 2018-09-24

**Authors:** Andrew J. Shoffstall, Melanie Ecker, Vindhya Danda, Alexandra Joshi-Imre, Allison Stiller, Marina Yu, Jennifer E. Paiz, Elizabeth Mancuso, Hillary W. Bedell, Walter E. Voit, Joseph J. Pancrazio, Jeffrey R. Capadona

**Affiliations:** 1 Department of Biomedical Engineering, Case Western Reserve University, Cleveland, OH, USA; andrew.shoffstall@case.edu (A.J.S.); mhy7@case.edu (M.Y.); jep141@case.edu (J.E.P.); mancuso.33@buckeyemail.osu.edu (E.M.); hillary.bedell1@gmail.com (H.W.B.); 2 Advanced Platform Technology Center, Rehabilitation Research and Development, Louis Stokes Cleveland Department of Veteran Affairs Medical Center, Cleveland, OH, USA; 3 Department of Materials Science and Engineering, The University of Texas at Dallas, Richardson, TX, USA; melanie.ecker@utdallas.edu (M.E.); vxd160030@utdallas.edu (V.D.); walter.voit@utdallas.edu (W.E.V.); 4 Center for Engineering Innovation, The University of Texas at Dallas, Richardson, TX, USA; alexandra.joshi-imre@utdallas.edu; 5 Department of Bioengineering, The University of Texas at Dallas, Richardson, TX, USA; axs169031@utdallas.edu (A.S.); joseph.pancrazio@utdallas.edu (J.J.P.); 6 Department of Mechanical Engineering, The University of Texas at Dallas, Richardson, TX, USA

**Keywords:** intracortical, microelectrodes, shape-memory-polymer, electrophysiology

## Abstract

Thiol-ene based shape memory polymers (SMPs) have been developed for use as intracortical microelectrode substrates. The unique chemistry provides precise control over the mechanical and thermal glass-transition properties. As a result, SMP substrates are stiff at room temperature, allowing for insertion into the brain without buckling and subsequently soften in response to body temperatures, reducing the mechanical mismatch between device and tissue. Since the surface chemistry of the materials can contribute significantly to the ultimate biocompatibility, as a first step in the characterization of our SMPs, we sought to isolate the biological response to the implanted material surface without regards to the softening mechanics. To accomplish this, we tightly controlled for bulk stiffness by comparing bare silicon ‘dummy’ devices to thickness-matched silicon devices dip-coated with SMP. The neuroinflammatory response was evaluated after devices were implanted in the rat cortex for 2 or 16 weeks. We observed no differences in the markers tested at either time point, except that astrocytic scarring was significantly reduced for the dip-coated implants at 16 weeks. The surface properties of non-softening thiol-ene SMP substrates appeared to be equally-tolerated and just as suitable as silicon for neural implant substrates for applications such as intracortical microelectrodes, laying the groundwork for future softer devices to improve upon the prototype device performance presented here.

## 1. Introduction

Intracortical microelectrodes are used for electrophysiology recordings from the brain in a number of applications across both basic neuroscience and rehabilitation [1,2,3,4]. The specific needs of the given application dictate how long the microelectrode must endure, what type of signal is required (e.g., single units versus local field potential), and the design of the electrode required for reaching the targeted location [5,6,7,8,9]. Unfortunately, the currently available implantable microelectrode arrays do not demonstrate long-term robustness, as evidenced by a gradual decline in the signal-to-noise ratio and ultimately a diminishing percentage of contacts that are able to record spiking behavior [10,11,12,13]. Therefore, there are many types of intracortical microelectrodes under development [3,4].

The failure mechanisms of recording microelectrodes are multifaceted and include a number of interrelated processes involving mechanical, material, and biological pathways [14,15,16,17,18]. Among these interrelated processes, the neuroinflammatory response is thought to play a central role in microelectrode failure. Prolonged neuroinflammation can cause a build-up of oxidative species that can promote neurodegeneration while also initiating the degradation of implanted materials, resulting in a positive feedback cycle [19,20]. Numerous materials-based and therapeutic strategies have the potential to intervene in several of the failure modes by combining mechanical strategies, bioactive coatings, and/or drug-eluting substrates [21].

To reduce the tissue response and combat chronic neurodegeneration around implanted intracortical microelectrodes, the neural engineering field has been increasingly moving toward smaller, softer materials and electrode designs [21,22,23]. Using soft polymer substrates, like polyimides [24,25,26] or Parylene-C [27], compliant devices appear to reduce the appearance of chronic inflammation in end-point histology [28,29,30]. Ultra-small concepts have also proven to work well for reaching superficial cortical targets of the brain [28,31,32,33]. However, such devices may not be compatible with implantation strategies for more difficult to access structures of the brain.

Thiol-ene and thiol-ene/acrylate shape memory polymers (SMPs) comprise a new class of substrate under development for neural interfaces [34,35]. Thiol-ene/acrylate acts as a versatile material that is stiff at room temperature and softens after implantation in response to body temperatures and fluid exposure. The softening effect can be as large as a transition from 1 GPa to 18 MPa [35]. The combination of thiol, alkene and acrylate monomers modulates the rubbery modulus and allows for the adjustment of the glass transition temperature via a composition ratio (i.e., relative concentrations of multivalent monomers from all three groups) [36,37,38]. Sterilization methods have been optimized [39], allowing for the first demonstration of acute recordings signals from the primary auditory cortex of rats [40]. While thiol-ene-based SMPs appear to be promising, thus far, there have not been robust analyses of the neuroinflammatory response elicited by their long-term implantation in the cortex.

The objective of the current study was to quantify the neuroinflammatory response to implanted SMP materials. Here, we chose to first compare our SMP to a bare silicon substrate similar to those used in commercially available planar microelectrodes. The goal was to first understand the biological response to the thiol-ene material itself, without the confounding variable of stiffness (and resulting differential tissue strains). To that end, the SMP material was dip-coated onto a silicon surface and compared to a size-matched bare silicon substrate. As a result, the bulk flexibility of the microelectrode was held consistent, while only the tissue-exposed surface varied. Given the similar size and stiffness, we hypothesized that stiff silicon microelectrodes dip-coated with a shape memory polymer would elicit a similar or reduced neuroinflammatory response compared to size-matched bare silicon microelectrodes after implantation into the rat cortex.

## 2. Materials and Methods

### 2.1. Study Design

Male Sprague Dawley rats (200–250 g, *n* = 11 per group) were implanted with either stiff silicon microelectrode probes dip-coated with shape memory polymer or size-matched bare silicon microelectrode probes. As performed previously by our group and others, microelectrode probes were implanted bilaterally (one in each hemisphere) and treated as independent of one another [41,42]. After implantation, animals were housed for 2 or 16 weeks, spanning the periods of initial and late-onset neurodegeneration [43,44]. Immunohistochemistry markers tested included neuronal density (NeuN), activated microglia (CD68), blood-brain barrier permeability (Immunoglobulin G (IgG)), and reactive astrocytes (glial fibrillary acidic protein (GFAP)) [20,43,45,46,47,48,49].

### 2.2. Device Fabrication and Sterilization

**Figure 1:**
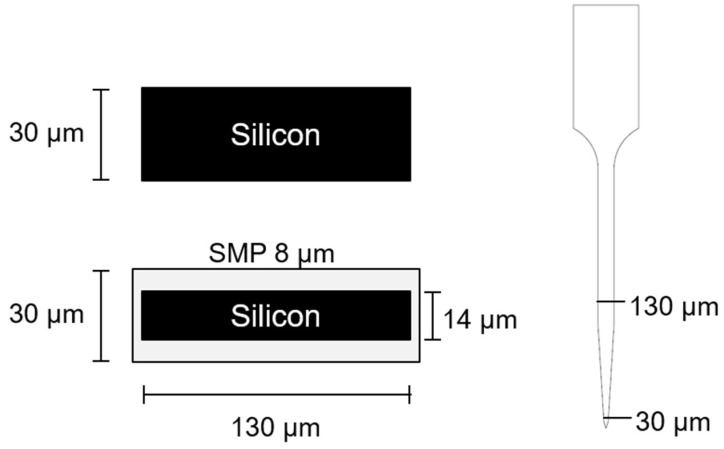
Probe design schematic. Cross-sectional dimensions of the silicon (**top**) and dip-coated (**bottom**) devices and view of the profile from the side (right). Here, 30 µm thick silicon wafers were used to fabricate the bare silicon probes whereas a 14 µm thick silicon wafer (after etching) was used to produce the dip-coating substrate so that the overall device thickness resulted as ~30 µm for both device types. Due to the photomasks used, the widths of the etched silicon devices were held constant so that the bare silicon probes were 130 µm in width and after coating, the dip-coated probes were slightly larger, ~135 µm, in width. The actual coating thickness varied slightly along the length of the probe as shown in Supplementary Figure S1.

Silicon ‘dummy’ probe devices, substrates without a recording functionality, were fabricated by a photolithographic process using a deep-reactive ion etching procedure as described below. In detail, silicon shanks of the desired thickness were fabricated from the appropriate SOI (silicon on insulator) wafer. These SOI wafers contained a device silicon layer and a buried oxide layer of 2 µm on top of 400 µm silicon handle layer. A hard mask of 2 µm thermal oxide was grown on these SOI wafers using a Tystar Diffusion/Oxidation Furnace. The wafers were then patterned using standard lithography techniques to yield the desired probe pattern. The thermal oxide hard mask and the device silicon layer were etched using CHF_3_/Ar plasma and a Bosch sequence (SF_6_/Ar and C_4_F_8_ plasmas) using a Plasma-Therm deep silicon etcher respectively. The buried oxide layer was used as the etch stop layer for the device silicon etch. The wafers were then soaked in solvent to remove the photoresist residues and in diluted 10:1 hydrofluoric acid overnight to lift-off the silicon shanks before they were triple rinsed in distilled water. The silicon shanks were further singulated from each other by breaking the tab that connects them using very fine metal tweezers under a microscope. The use of two different thickness of silicon wafers allowed us to generate devices that were either 30 µm or 14 µm thick (25 µm prior to etching). The latter devices were then modified with a dip-coating process to add a thiol-ene shape memory polymer to generate a nearly equivalent ~30 µm thick SMP dip-coated device (Figure 1).

SMP pre-polymer solution was prepared as described previously [39]. The material is characterized by a glass transition temperature (*T*_g_) of 45 °C in the dry state, and a *T*_g_ of 30 °C after being soaked in phosphate buffered saline (PBS) at 37 °C for at least 30 min. The storage modulus *E*′ of the materials decreases from 1.7 GPa (dry) to 20 MPa (wet) due to plasticization effects. The monomer ratios were 50 mol% 1,3,5-Triallyl-1,3,5-triazine-2,4,6(1H,3H,5H)-trione (TATATO), 45 mol% trimethylolpropane tris(3-mercaptopropionate) (TMTMP), and 5 mol% Tris[2-mercaptopropionyloxy)ethyl] isocyanurate (TMICN). All monomers were mixed with 0.1 wt% of photoinitiator 2,2-dimethoxy-2-phenylacetophenone (DMPA) before they were used for dip coating.

The silicon shanks were individually mounted on ethylene oxide indicator tape for handling purposes. To enhance the SMP adhesion, bare silicon shanks were subjected to a surface treatment prior to the dip-coating which included a roughening step via O_2_/Ar plasma in a March reactive ion etching (RIE) system for 16 min at 200 mT/50 W followed by SF_6_ plasma treatments at 120 mT/100 W for 5 min in a Technics RIE system on both sides. The surface-treated silicon shanks were then held with tweezers and dipped manually at an average speed of 7–13 mm·s^−1^ (assessed by videotaping) into the pre-polymer solution. The viscosity (h) of the thiol-ene pre-polymer solution was 0.18 ± 0.1 Pa·s (TA discovery HR3 rheometer (TA Instruments, New Castle, DE, USA), sweep from 1 to 1000 s^−1^). The SMP solution was prepared immediately before dip-coating. The coated pre-polymer was cured on all sides using a 365 nm handheld UV gun for about 30 s. The probes were inspected under an optical microscope to ensure the shank was coated successfully (i.e., showing full polymer coverage and no beads). Only sufficiently coated probes were selected and fully cured for 1 h in a cross-linking chamber (UVP CL-1000 (UVP, LLC, Upland, CA, USA) with five overhead bulbs) followed by an overnight post-cure at 120 °C under vacuum. To verify the thickness of the SMP layer and to ensure evenly coated surfaces, the fully cured samples were investigated using scanning electron microscope (SEM) (Zeiss Supra 40 and Zeiss EVO LS 15 Scanning Electron Microscopes, Zeiss, Inc., Oberkochen, Germany). SEM parameters included: EHT (accelerating voltage) between 0.5 kV and 5.0 kV and various magnifications. Individual parameters are displayed at the bottoms of the SEM images. The contact angle of water for the SMP material was ~70° while the silicon had a contact angle of ~40°.

Devices were sterilized using ethylene oxide (EtO) as previously described [39]. Briefly, the devices were loaded into a liner bag along with gas indicator tape and a glass ampoule containing 18 g of liquid EtO before it was sealed using Velcro wrap and placed into the ethylene oxide sterilizer (AN 74i, Anprolene, Andersen Sterilizers Inc., Haw River, NC, USA). The sterilization cycle at atmospheric pressure lasted for 24 h followed by a 2 h purge/aeration. To remove any residual EtO from the samples, they were subjected to an addition degassing for 72 h at 37 °C under vacuum. To validate the effectiveness of this sterilization method, we have previously performed residual endotoxin testing on pre- and post-sterilization materials as well as a host of mechanical testing (e.g., dynamic mechanical analysis (DMA)) to ensure that the mechanical properties were not adversely impacted in the described process [39].

### 2.3. Device Implantation

All procedures were reviewed and approved by the Louis Stokes Cleveland Department of Veterans Affairs Institutional Animal Care and Use Committee. Sprague Dawley rats were anesthetized (3–5%) and kept under anesthesia (1–3%) using an isoflurane vaporizer to maintain a surgical plane of anesthesia. Once anesthetized, eye lubricant was applied and the fur on the scalp was shaved and cleaned. Prior to surgery, the rats received 16 mg/kg cefazolin and 1 mg/kg meloxicam subcutaneously as a prophylactic antibiotic and analgesic, respectively. Additionally, a single dose of 0.2 mL of 0.25% bupivacaine (local anesthetic) was administered subcutaneously at the incision site. The surgical site was cleaned in triplicate with betadine followed by isopropyl alcohol scrubs. Surgery was performed under an operating microscope. Craniotomies were performed carefully with a combination of intermittent pausing and saline application, to prevent overheating from drilling [50]. A sterile ruler and forceps were used to mark the area to be drilled, 2 mm lateral to midline, 3 mm posterior to bregma (corresponding to a region of the sensory cortex). Removal of the final thinned bone flap was performed with ultrafine rongeurs to prevent incidental mechanical damage to the brain from the drill tip. After careful reflection of the dura, microelectrodes were implanted ~2 mm deep by hand using micro-forceps, avoiding superficially visible vasculature. Kwik-Cast was applied to cover the craniotomy and allowed to cure, followed by application of cold-cure dental acrylic to build up a stable cement base around the implant. Given the low profile of the dummy probe implants (e.g., as compared to functional recording microelectrodes that require an exposed head-stage), the skin was sutured together and treated with a non-prescription triple-antibiotic cream. A post-operative analgesic was provided for 2 days following implantation (1 mg/kg meloxicam q.d.) and post-operative prophylactic antibiotics were provided for 1 day following implantation (16 mg/kg cefazolin, b.i.d.). There were no complications with post-operative infection or observations of overtly unmanaged pain from the procedure.

### 2.4. Tissue Extraction and Preparation

At the pre-determined end points (2 or 16 weeks), animals were perfused transcardially under deep anesthesia to prepare the tissue for histological processing. After achieving a deep plane of anesthesia, using a ketamine/xylazine cocktail (80 mg/kg and 10 mg/kg respectively), rat aortas were cannulated with a gavage needle via an incision in the left ventricle and connected to a perfusion pump. Phosphate buffered saline (1×) was perfused until the fluid exiting the excised vena cava/right atrium appeared clear. Tissue was then fixed by perfusion with ~200 mL of 4% *w*/*v* paraformaldehyde solution. The tissue was post-fixed in 4% *w*/*v* paraformaldehyde solution overnight. After careful extraction, brains were subsequently cryoprotected with a gradient of sucrose (with 0.1% sodium azide) from 10 % to 30% *w*/*v* and frozen in OCT (Opjmal Cu ng. Temperature) blocks and stored at −80 °C until sectioning. Tissue sections, 20 µm thick, were generated on a cryostat and were collected on Fisherbrand ‘Superfrost Plus’ glass slides.

### 2.5. Quantification of Immunohistochemistry

Immunohistochemistry was performed as previously described for neuronal density (NeuN), activated microglia (CD68), blood-brain barrier permeability (IgG), and astrocytes (GFAP) [45,51]. To account for the known variation of the histological response along the depth of the implant, horizontal (transverse) slices were collected and compared at an array of locations spanning randomized depths of 500–1500 microns. Stained slides were imaged using a 10× objective on an AxioObserver Z1 (Zeiss, Inc.) and AxioCam MRm (Zeiss Inc.). All images except those stained for NeuN were analyzed using SECOND (version 030918, MathWorks, Inc., Natick, MA, USA), a custom MATLAB program developed to analyze fluorescent intensity profiles around the electrode [52]. In summary, the void in the tissue left by the explanted microelectrode was manually defined by tracing each image on-screen. The area defined by this tracing was collected and used to tabulate the explanted hole size. Fluorescent intensity was then tabulated by the program in expanding concentric contours around the microelectrode-tissue interface edge. To quantify neuron populations around the implant site, the number of neurons in each ring was manually counted to obtain the number of neurons per area for each radial distance [19,43]. In each case a normalized metric was generated, where the average intensity for a given concentric bin was divided by a concentric bin far enough away from the microelectrode implant that the neuroinflammatory response was minimal: 600–650 µm for intensity-based measures (IgG, GFAP, CD68), and 250–300 µm for count-based measures (NeuN).

Statistics were calculated in Minitab 18 (State College, PA, USA). The continuous outcome measures, including neuronal density, captured at endpoint histology were evaluated to compare inter-group differences for individual distance buckets (i.e., 0–50 µm, 50–100 µm,) using two-sample *t*-tests, with a significance at level *p* < 0.05. Intensity-based histological measures (GFAP, CD68, IgG) were analyzed using previously established quantification methods [43,46], wherein the fluorescent intensity was plotted as a function of distance from the electrode surface and the statistical outcome was the area-under-the-curve, corresponding to the level of overall tissue response for a given stain. Similarly, inter-group differences for individual distance buckets were calculated by two-sample *t*-tests, with a significance at level *p* < 0.05.

### 2.6. Characterization

#### 2.6.1. Dip-Coating Silicon ‘Dummy’ Microelectrodes with Thiol-ene Polymer

**Figure 2:**
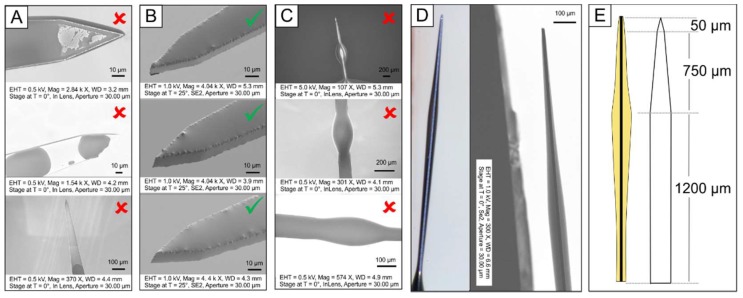
Characterization of silicon ‘dummy’ microelectrode with thiol-ene polymer. Dip coating of 25 µm thick microelectrodes with a uniform layer of shape memory polymer (SMP) to generate approximately 30 µm thick coated devices; (**A**) the polymer detached before surface modification of silicon probes, (**B**) nicely coated the silicon shanks after surface modification. (**C**) In some cases, the coating would form ‘beads’ due to slow removal. Checkmarks and crosses indicate whether probes were used for in vivo studies or not. (**D**) Optical and scanning electron microscope (SEM) images in the side view to assess the thickness of the coating, and (**E**) schematic drawing of coating thickness with respect to the shank geometry.

Dummy microelectrodes were successfully dip-coated with a thiol-ene polymer (Figure 2). Multiple parameters appeared to influence the coating thickness and ability to form a uniform layer, especially the surface of the silicon shanks and the rate of removal from the polymer solution. If the silicon shanks were unmodified, the SMP did not adhere well to the surface (Figure 2A). However, after applying a surface treatment to the bare silicon shanks (O_2_/Ar and SF_6_ plasma), the shanks could be uniformly coated with SMP (Figure 2B). The viscosity of the pre-polymer solution, *h* = 0.18 ± 0.1 Pa·s, was set by the previously defined composition of monomers. A change in the composition would result in a change of thermomechanical properties and was therefore not desired. Another way to change the viscosity of the solution would be by the addition of solvent. However, the addition of solvent would alter the curing kinetics and cross-link density and thus the thermomechanical properties as well. Therefore, only the removal speed could be varied to alter the surface coating. An average speed of 7 to 13 mm·s^−1^ of manual dip-coating turned out to result in sufficient coatings (Figure 2B). In some cases, more dominant at lower speeds, beading of the coating was visible along the microelectrode shank (Figure 2C). Only probes which showed full coating and no visible beads were used for the in vivo study.

Dip coating quality and uniformity were approximated using optical microscopy and SEM on several representative devices. We found that the coating profile was consistent across various probes. The thickness of the coating, however, had no uniform thickness throughout the length of the shanks (Figure 2D). The tip of the probes had a very thin layer of polymer (less than 1 µm), whereas the rest of the probe had a layer thickness of about 10 to 30 µm on the top and bottom, respectively. The thickest coating was consistently found at the part of the probes where the shank started to narrow down about 850 µm distance from the tip (Figure 2E). This can be explained by the dipping process. The pre-polymer solution was flowing down the probes due to gravity before they were cured and accumulated at the abovementioned part of the probe due to an abrupt change of geometrical surface area. After the surface modification of the bare silicon shank, which included reactive ion etching, the thickness of the shanks was reduced from 25 µm to 14 µm. The averaged thickness of the SMP coating across the surface was approximately 8 µm on either side, which adds up to an overall probe thickness of about 30 µm. Even if the coating had no consistent thickness throughout the probe, the overall probe volume, and with that the averaged footprint of the implanted part of the dip-coated probes, was still similar to that of bare silicon probes.

#### 2.6.2. Durability of Dip Coated Probes

**Figure 3:**
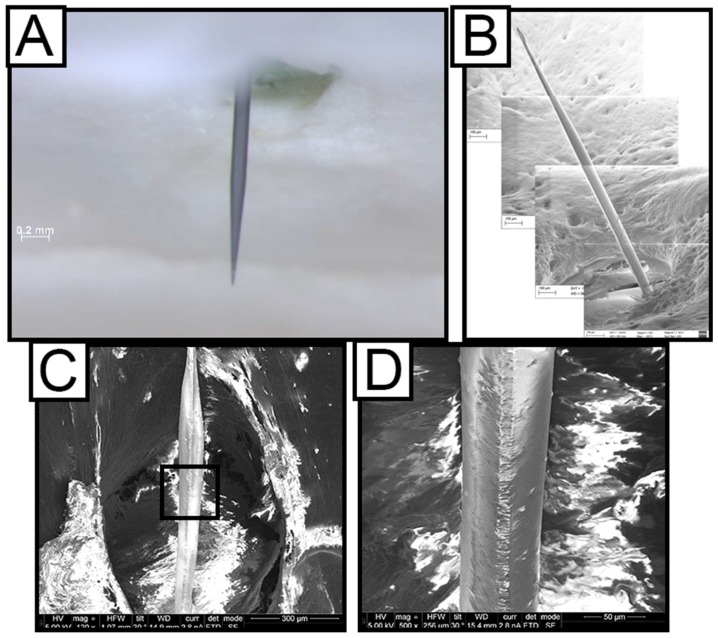
Ex vivo Characterization of coating stability. Dip-coated probes inside the skull with all tissue removed captured using optical microscopy (**A**) and SEM (**B**) showing that the SMP coating of the silicon shanks is still intact after two weeks. (**C**) Side-view SEM image of a dip-coated probe, explanted after 16 weeks in the rat cortex, showing the SMP coating intact. Black rectangle inset is blown up further in (**D**).

The thiol-ene formulation used here was stable under physiological conditions for up to 13 months without any signs of hydrolytic degradation (paper under review). In order to directly test the dip-coating stability after in vivo implantation, explanted polymer coated silicon probes were investigated after 2 weeks (Figure 3A,B) and 16 weeks of dwelling in the rat cortex (Figure 3C,D). The coating of the probes was investigated before and after aging by means of SEM imaging. It was found that the coating was stable and did not show any signs of degradation over the course of implantation. The coating looked intact after being implanted into rat cortex for 2 weeks and 16 weeks, as demonstrated by the representative SEM images in Figure 3. In order to further assess the durability of the SMP coating and its adhesion to the silicon shanks, the dip-coated probes were further tested in vitro under accelerated aging conditions. The dip-coated probes were mounted onto the cap of a glass jar and were immersed into phosphate buffered saline (PBS) at 57 °C for a minimum of 84 h. While the surfaces of the probes became rougher, they remained visibly intact under SEM imaging (Supplementary Figure S2).

#### 2.6.3. Tissue Void from Device Explantation

**Figure 4:**
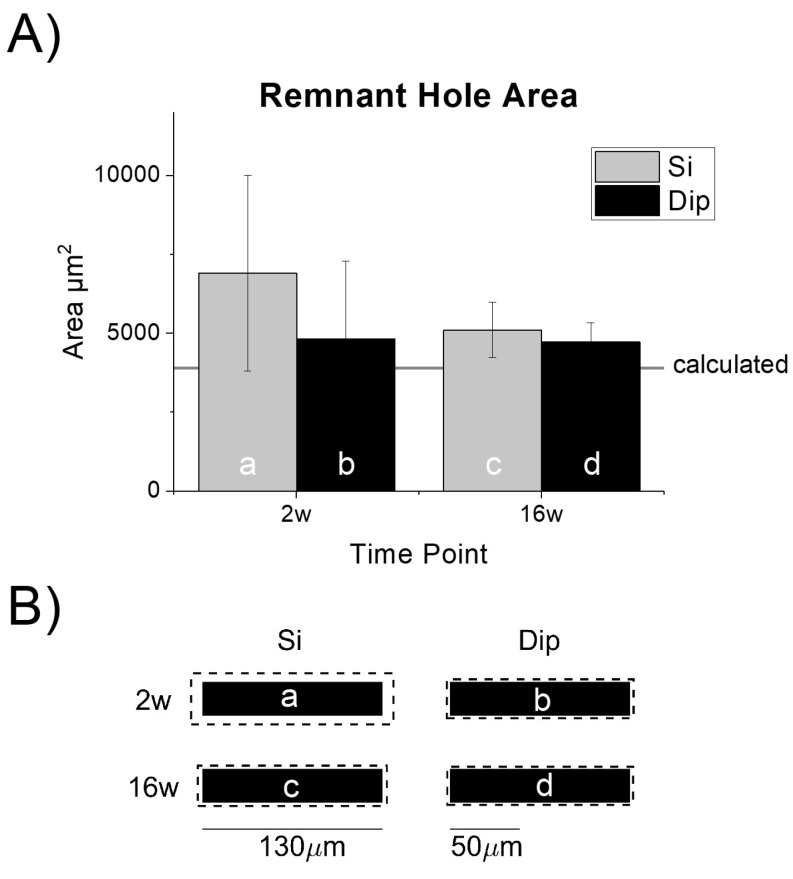
Characterization of the remnant tissue hole after explantation and ‘dummy’ probe device dimensions. (**A**) Remnant hole size after probe extraction was consistent (no statistically significant differences) across both implant types. The hole was slightly larger than the theoretical cross-sectional area denoted by the horizontal line; *n* = 9 (Si-2w), 10 (Si-16w), 10 (dip-2w), 10 (Dip-16w). (**B**) Mean explanted hole size (dashed line) drawn in relative scale to the actual device dimensions. The letters correspond with the matching bar in the chart shown in (**A**). The 130 µm scale bar is shown to provide context for the microelectrode dummy probe width. The 50 µm scale bar provides context for the analysis of bucket widths for the histological analysis. Tissue responses can extend several hundred microns away from the tissue-device interface.

To verify that the cross-sectional area was held consistent between the two types of implants, we quantified the remnant hole in the tissue after device explantation. As expected, the hole sizes were similar to, but slightly larger than, the cross-sectional area of the actual probe devices (~3900 µm^2^, Figure 4A,B). The difference in the hole to device size could be due to a thin layer of tissue remaining adhered to the microelectrode, or any slicing (edge) artifact. Interestingly, the remnant tissue hole size variability appeared to be higher at 2 weeks compared to 16 weeks and may have resulted from a looser, more immature scar or increased edema at that time.

As described above, the size of the dip-coated probe devices was controlled by applying a ~8 µm thick layer of SMP to a bare silicon device with 14 µm thickness to achieve an approximately 30 µm overall thickness (Figure 1). Compared to the actual device sizes, the increased hole diameters appeared to be marginal (Figure 4B). In the largest group, two-week silicon implants (‘2w-Si’), the equivalent mean increase in radius was calculated to be ~16 microns (Figure 4B).

## 3. Results

### 3.1. Endpoint Histological Analysis

#### 3.1.1. Astrocyte Response

Astrocytes, a glial cell, play a number of important roles in the brain, including contributing to the blood-brain barrier [16,53]. They react to injury and foreign implanted materials in the brain by changing morphology, migrating toward the implant, and expressing or upregulating the expression of a host of proteins, including glial fibrillary acidic protein (GFAP) [18,54].

**Figure 5:**
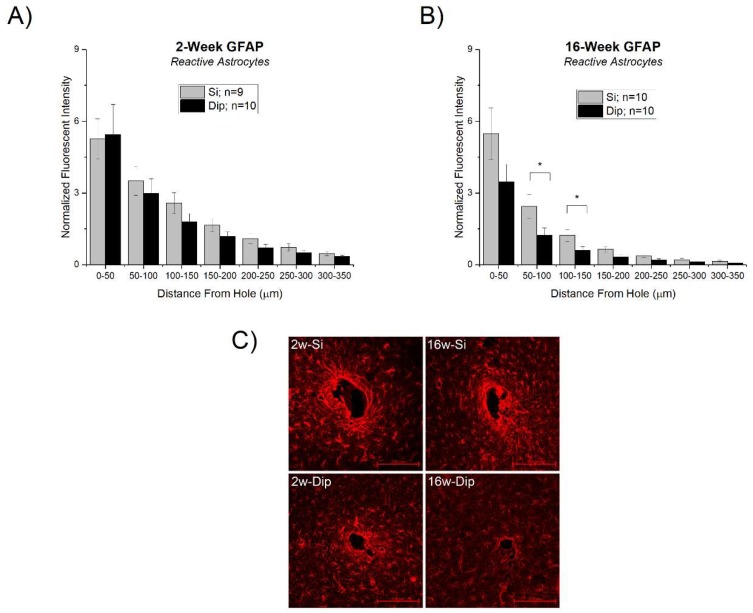
Astrocytic response to silicon vs. SMP dip-coated implants. (**A**) Astrocytic scarring at 2 weeks and (**B**) 16 weeks. There were significant differences between the silicon and dip-coated glial fibrillary acidic protein (GFAP) response at 16 weeks, specifically at bucketed distances 50–100 µm and 100–150 µm from the hole. There were no differences between the groups at 2 weeks or any other regions from the hole at 16 weeks post-implantation. (**C**) Representative images of the GFAP staining results with 200 µm scale bars in the bottom right-hand corner.

While there were no significant differences between silicon and SMP dip-coated devices at 2 weeks (Figure 5A), there was a significantly lower response for dip-coated implants at 16 weeks compared to the bare silicon control. Specifically, the statistically significant differences were in the concentric ranges 50–100 µm and 100–150 µm from the hole remaining after device extraction (Figure 5B).

#### 3.1.2. Activation of Microglia and Macrophages and Blood-Brain Barrier Permeability

**Figure 6:**
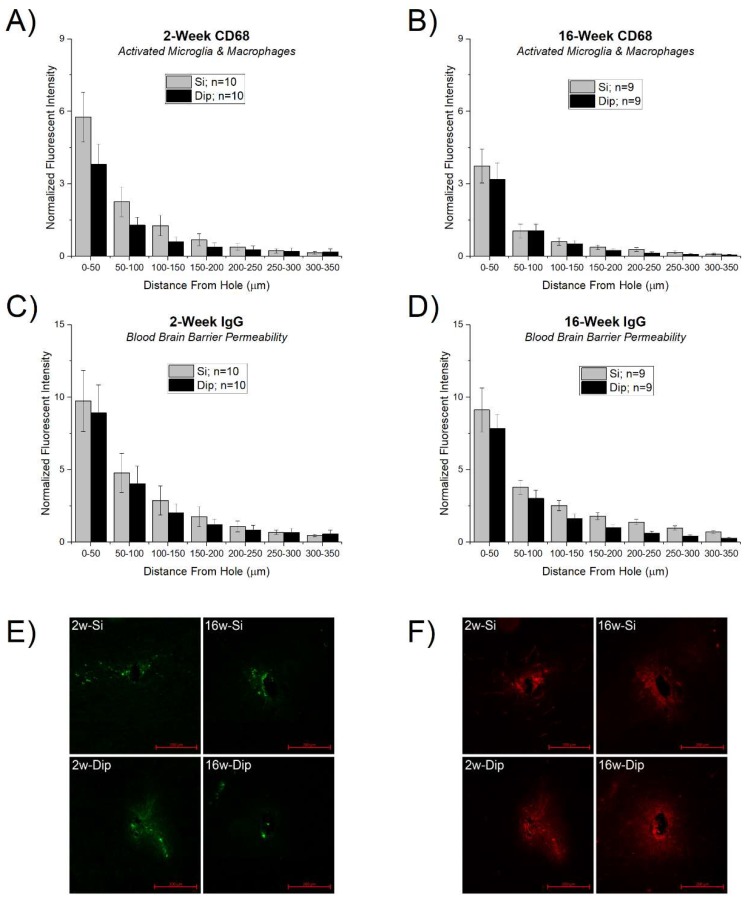
Microglia and BBB response to silicon vs. SMP dip-coated implants. Activated macrophages and microglia (CD68) at (**A**) 2 weeks and (**B**) 16 weeks. Blood-brain barrier (BBB) permeability marked by immunoglobulin G (IgG) staining at (**C**) 2 weeks and (**D**) 16 weeks after microelectrode implantation. There were no differences in either stain between each probe type for either time point tested. (**E**,**F**) Representative images of the CD68 (**E**) and IgG (**F**) staining results with 200 µm scale bars in the bottom right-hand corner.

CD68 is a marker of activated microglia and macrophages and has been associated with heightened neuroinflammatory responses to implanted foreign materials in the brain [55,56,57,58]. IgG is a blood immunoglobulin protein not normally found in the brain and is therefore commonly used as a marker for blood-brain barrier permeability [59]. All the stains followed a typical decay profile with the largest expression at the explant hole edge, returning to a baseline response within a few hundred microns. While overall there was a trend of a reduced response in both CD68 (Figure 6A,B) and IgG (Figure 6C,D), there were no statistically significant differences between any of the silicon and SMP dip-coated implants for either stain at each time point.

#### 3.1.3. Neuronal Density

**Figure 7:**
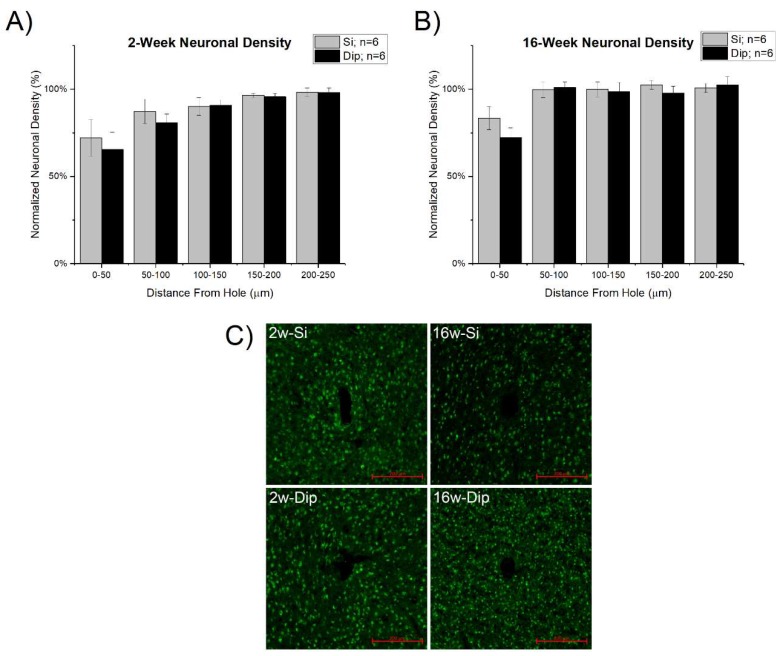
Neuronal density (NeuN staining) at (**A**) 2 weeks and (**B**) 16 weeks after microelectrode implantation. There were no significant differences between either material group, silicon vs dip-coated, at the two time points tested. (**C**) Representative images of the NeuN staining results with 200 µm scale bars in the bottom right-hand corner.

Insertion surgery and the subsequent neuroinflammatory response is thought to contribute to neuronal loss near the microelectrode interface [60]. In our studies, a decrease in neuronal density near the explanted microelectrode site was observed both at 2 weeks and 16 weeks for both probe types (Figure 7). For both probe types, neuronal densities returned to background levels by 200–250 μm at 2 weeks post-implantation and by 50–100 μm at 16 weeks post-implantation. There were no statistically significant differences between the silicon and dip-coated implants.

## 4. Discussion

Thiol-ene and thiol-ene/acrylate shape memory polymers (SMP) are under development for use as an implanted microelectrode substrate [35]. The materials are advantageous owing to their unique and highly tunable chemistry, the potential for reproducible manufacturing, and ability to soften after implantation in the brain [40]. We have previously demonstrated that intracortical probes that reduce their modulus after implantation into the brain significantly reduce the resulting neuroinflammatory response [45,61,62,63], likely due to the reduction in tissue strain and micro-motion [64]. We have also shown that the protein, cellular, and tissue responses to synthetic materials are highly correlated to the surface chemistry [65,66,67,68,69]. Therefore, in order to disentangle the potential differential effects on the biological response from both the new material chemistry and the unique mechanics, in this study, we first sought to hold the bulk mechanics as constant as possible while only varying the surface exposed to the brain tissue after implantation. By dip-coating SMP onto the surface of silicon probe devices, we were able to compare the biological response to implanted thiol-ene polymer materials without regards to their bulk stiffness or flexibility.

The only marker with statistically significant results was in the astrocytic response after 16 weeks of implantation, where the dip-coated silicon devices exhibited a significantly lower response than the bare silicon control devices at distances of 50–150 µm from the implant surface (Figure 5B). We found no statistically significant differences between the two implanted probes with regards to microglia and macrophage activation, blood-brain barrier permeability, or neuronal density. While the surface mechanics of the dip-coating layer may have played a minor role, we have shown before that the overall flexibility of the devices is driven by the underlying silicon [62]. Several studies have investigated the effects of substrate stiffness (modulus) on the astrocytic response, both in vitro [70,71] and in vivo [70,72], showing an enhanced response around stiff materials (~10 kPa) compared to softer materials (~100 Pa). While consistent with our overall observations of a decrease in activated astrocytes proximal to the implant, it is important to note that the modulus of the SMP coating here was at least twice as stiff as the “stiffer” substrates in each of the mentioned studies, with “soft” substrates often an order(s) of magnitude softer than the SMP reported here.

As the neuroinflammatory events surrounding the implanted microelectrode are constantly changing with respect to glial cell density, biochemical environment, neuron viability, and blood-brain barrier leakage [13,73,74], it is important to consider several time points. Here, we chose 2 weeks and 16 weeks, as they correspond with both early-onset and late-stage neurodegeneration [43]. Upon implantation, microelectrodes immediately disrupt brain tissue and neurovasculature, initiating a multi-phasic inflammatory response [3,43]. The acute response plateaus within the first few weeks of implantation and inflammation thereafter is driven in a chronic state by various mechanisms, including the fibrotic glial scar formation, microglial and macrophage activation, free radical oxidation and chronic dysfunction of the blood-brain barrier [43,49,75,76].

In the current study, the astrocytic response was observed to be greater for the non-coated silicon at the 16 week time point but not the 2 week time point. Since GFAP expression was only greater at the later-stage time point, our results suggest that the main differences appear after the normal wound-healing response has subsided. The overall magnitude of the response to the silicon implants at 16 weeks was similar to that of the silicon and dip-coated material at 2 weeks. Therefore, the increased astrocyte response for silicon versus the dip-coated at 16 weeks appears as a prolonged state of activation phase (or inability to ‘de-activate’) rather than the increased initial magnitude of astrocyte activation. Furthermore, differences were not observed in any of the other markers, including microglial/macrophage activation, blood-brain barrier permeability, or neuronal density, suggesting that the different response may be uniquely centered around astrocytes (at least of the markers tested). Increased astrocytic scarring has been associated with negatively impacting signal quality, either through directly increasing tissue impedance, or through physical separation and increased distance between the microelectrode contacts and viable neurons [77]. Results demonstrating that the SMP material exhibits a reduction in GFAP expression at 16 weeks are promising and deserve further exploration in future recording studies.

The SMP polymer formulation used here demonstrated a slightly higher contact angle (more hydrophobic) compared to bare silicon. We have previously demonstrated that slight changes in contact angle measurements can have profound impacts on the nature of protein adsorption and the resulting cell adhesion [68]. Upon initial observation, the higher contact angle (more hydrophobic) SMP material would have been thought to lend itself to a greater adsorption of proteins that promote the development of the glial scar. On the contrary, we found less GFAP expression for the SMP group compared to bare silicon at 16 weeks. It is possible that the dip-coated surface may preferentially attract a different composition or conformation of adsorbed proteins that does not react with astrocytes as strongly. Similar results have been demonstrated with other coating approaches to microelectrode substrates.

By comparison, Lee found that coating planar silicon microelectrodes with polyethylene glycol (PEG) previously demonstrated no effect on the foreign body response [41]. Considering that PEG is highly hydrophilic, this is somewhat contradictory to the expected results. However, as noted by the authors of the paper, it is unlikely the PEG coatings remained intact over the course of the entire 4 week study. Considering the work reported by Lee, and our current study together, it is unclear if a high degree of hydrophobicity alone is enough to influence the long-term foreign body response.

Future studies will investigate the recording performance and neuroinflammatory response to probes made entirely from the SMP materials. Preliminary experiments with dummy (non-functional) SMP probes have suggested that stereotactic insertion of the SMP microelectrodes will be challenging. Due to their thin and flexible nature, many of the fabricated SMP devices have a curved surface that prevents their successful insertion using stereotactic methods. The probes bend, deflect, or buckle at a relatively high frequency. Since the current study was planned to be compared to a study of microelectrodes made completely of the SMP material, all implantations were performed by-hand to allow for cross-comparability between the experimental conditions. However, it is likely that the error injected into the study is minimal compared to other experimental confounds. We have previously compared the neuroinflammatory response of microelectrodes implanted by either stereotactic or by-hand method and found the variability of the histological responses to be negligible compared the larger subject-to-subject variability inherent with both methods (Supplementary Figure S3). While the magnitude of the impact may be debated, the discrepancy must be noted as a known limitation of the present study.

In conclusion, thiol-ene and thiol-ene/acrylate shape memory polymers are currently under development for a wide range of neural implant applications, including intracortical microelectrodes, spinal cord stimulators, sciatic-tibial-sural ‘Y’ electrodes, longitudinal intrafascicular electrodes, and self-coiling nerve cuff electrodes [22,35,40,78,79,80]. Here, our initial study sought to investigate the foreign body response to the material in absence of its mechanical softening properties so that as many extra and potentially confounding variables as possible could be eliminated. From the results of the current study, we can conclude that the thiol-ene polymer material performs similar to, or better than, bare silicon in terms of the biological markers tested over 16 weeks of implantation in the rat cortex. It is well documented that silicon-based microelectrodes, such as the controls used here, are not a long-term solution for intracortical microelectrodes. Therefore, the fact that our thiol-ene shape memory polymer merely performs as well is not overly inspiring. However, the anticipated advantage of the thiol-ene shape memory polymer system is in the mechanical softening, which was not exploited here. Therefore, at worst, our SMP is adequate as an intracortical microelectrode substrate. Additional studies are required to test how the mechanics (dynamically softening after implantation) will affect the neuroinflammatory response.

## Supplementary Materials

The following are available online at http://www.mdpi.com/2072-666X/9/10/486/s1, Figure S1: SEM measurement of dip-coating thickness along the length of the probe, Figure S2: Accelerated aging representative SEM, Figure S3: Comparing neuronal density around stereotactically and hand inserted Michigan-style microelectrodes 16 weeks post implantation.
