# Supplementary material for: Characterization of the Neuroinflammatory Response to Thiol-ene Shape Memory Polymer Coated Intracortical Microelectrodes
Source: Micromachines (Basel). 2018 Sep 24;9(10):486. doi: 10.3390/mi9100486 (PMC6215215; doi:10.3390/mi9100486)
Supplement: Supplementary file 1 [file micromachines-09-00486-s001.pdf]

Supplementary Materials: Characterization of the Neuroinflammatory Response to Thiol-ene Shape Memory Polymer Coated Intracortical Microelectrodes

Andrew J. Shoffstall, Melanie Ecker, Vindhya Danda, Alexandra Joshi-Imre, Allison Stiller, Marina Yu, Jennifer E. Paiz, Elizabeth Mancuso, Hillary W. Bedell, Walter E. Voit, Joseph J. Pancrazio and Jeffrey R. Capadona

1. Supplementary Results and Discussion

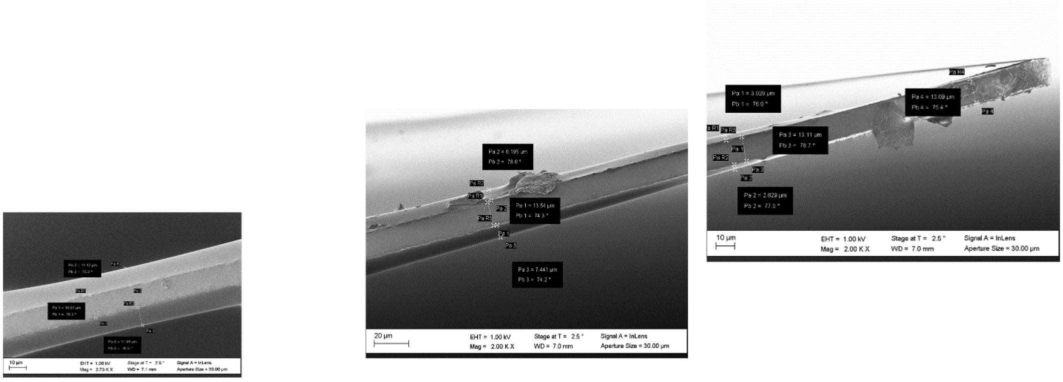

(a)

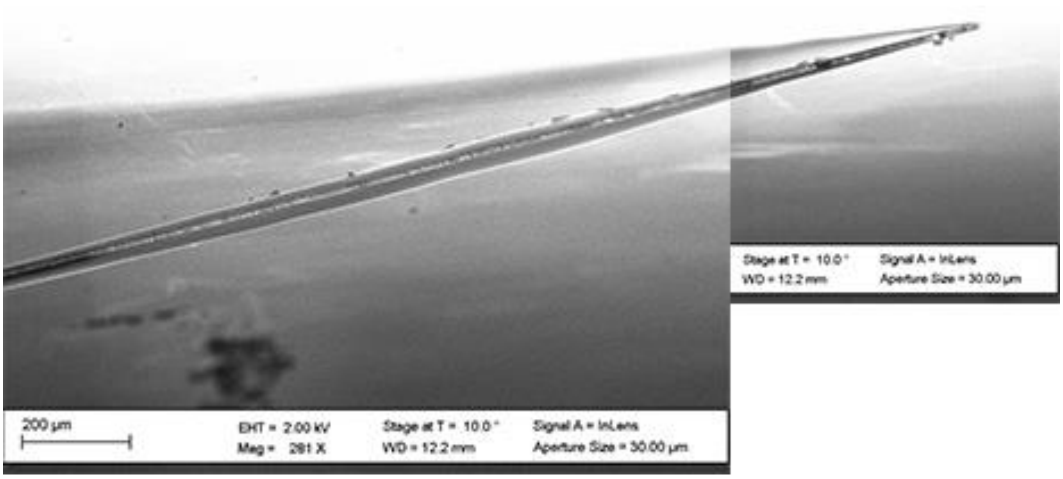

(b)

Figure S1. Scanning electron microscope (SEM) measurement of dip-coating thickness along the length of the probe. (**a**) set of 3 paneled images at the top show measurements of the coating thickness near the tip of the probe (magnification 2000×), and (**b**) set of 2 paneled images on the bottom at lower magnification. The coating can be seen as thinner toward the tip (right) and thicker toward the base (left).

Scanning electron microscope (SEM) images were taken of the dip-coated probes immediately after the curing process was complete. The images show the thickness of the coating varies over the length of the probe. The mean thickness along the length is ~8 um (range 2–24), (Figure S1).

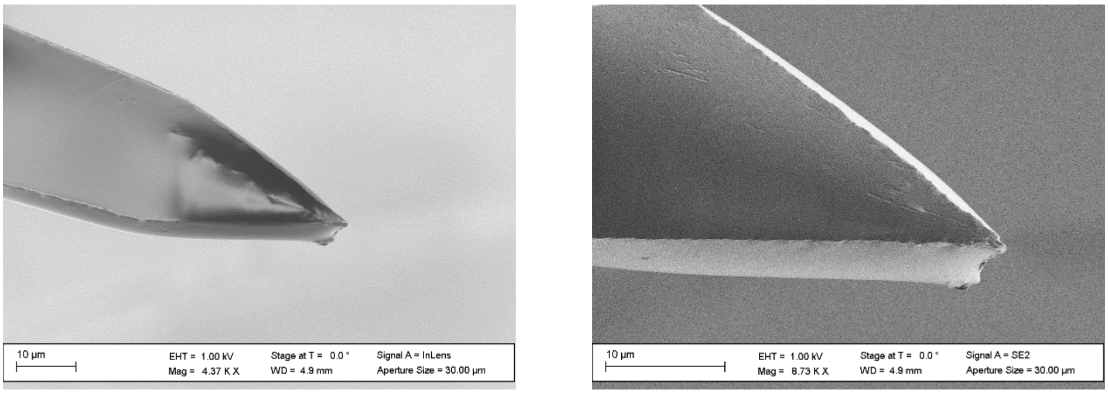

(a)

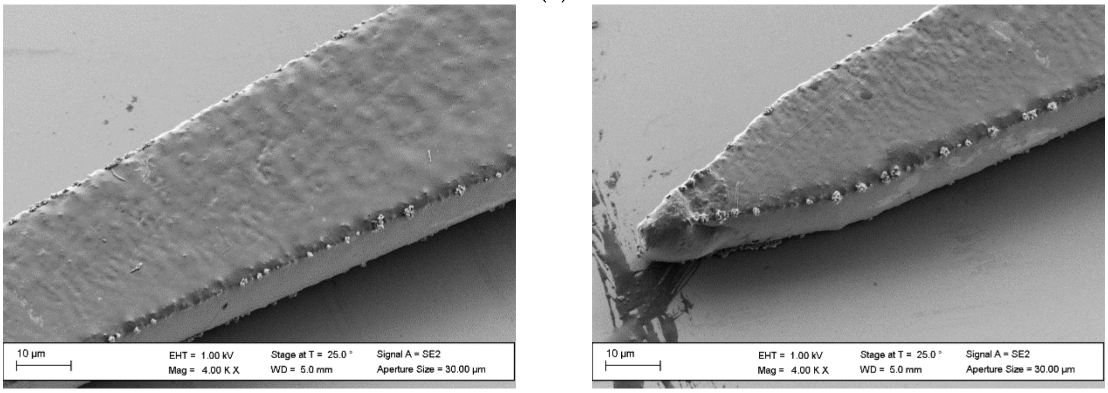

(b)

Figure S2. Accelerated aging representative SEM. Compared to the pre-aged sample (**a**), the aged probe surface (**b**) appears rough but intact.

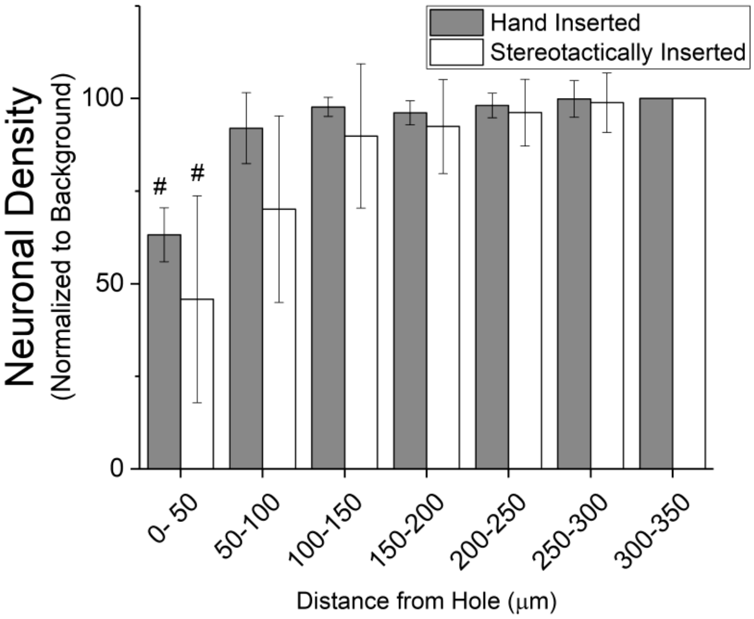

Figure S3. Comparing neuronal density around stereotactically and hand inserted Michigan-style microelectrodes 16 weeks post implantation. Neuronal density evaluated as $NeuN^+$ cells with respect to distance from the explanted microelectrode hole (µm). # Denotes significant difference from background neuronal density. ($n = 9$ for each condition).

Stereotactically inserted probes have more variance than hand inserted probes in this comparison study (Figure S2). Thus, using stereotactically implanted electrodes do not reduce the variability of the outcome examined—neuronal density at the electrode-tissue interface. This suggests that method of implanting single shank Michigan-style probes is not a strong determinant

in the variability of the inflammatory response and resulting neuronal death seen within a single group. Rather, like Kozai suggests, the variability in inflammation seen is a result of implant location relative to large blood vessels; if the electrode severs large vessels rather than small capillaries, more inflammation will occur [1]. One limitation to this comparison however, is that the stereotactically inserted probes were implanted in mice who ended up having large headcaps and went through twice-weekly recording sessions which included clipping the headstage on and off. It is important to note that the animals with the probes inserted by hand were handled very minimally throughout the 16 weeks post implantation. The additional motion and possible strain on the tissue surrounding the implant is confounded with the results.

2. Supplementary Methods

Male and female C57/BL6 (strain #000664) mice between 8–12 weeks of age were used for surgery. All animal practices were performed in a class II sterile hood using microisolator techniques. All procedures and animal care practices were approved by and comply with the Case Western Reserve University Institutional Animal Care and Use Committee.

For stereotaxic insertion, single shank, 16 channel Michigan style electrodes with iridium contact sites (A16-3mm-100-50-177-Z16) (3 mm × 15 μm × 123 μm) from NeuroNexus were implanted into the primary motor cortex ($n = 9$). The electrode was inserted into the cortex using a micromanipulator at a rate of approximately 10 μm/sec in 50 μm increments. Following the electrode implantation, silicone elastomer was used to seal the craniotomy and self-curing dental acrylic was used to secure the electrode connector forming a headcap. Awake electrophysiological recordings were conducted twice a week for 16 weeks on these animals.

For probes inserted by hand ($n = 9$), single shank, Michigan style silicon probes (3 mm × 15 μm × 123 μm) were implanted by hand into the primary motor cortex. Following probe implantation, silicone elastomer was used to seal the craniotomy and UV-cured dental acrylic was used to tether the probe to the skull. The incision site was then sutured closed using 5-0 monofilament polypropylene suture.

The following steps are consistent for animals with electrodes implanted by hand and electrodes implanted stereotactically. Sixteen weeks post implantation, after injection of a Ketamine/Xylazine cocktail, mice were then transcardially perfused with phosphate buffered saline then 4% paraformaldehyde (PFA) to fix the tissue. Following perfusion, the mouse heads were post-fixed for an additional two days in 4% PFA at 4 °C. After complete fixation, brains were then extracted and equilibrated in 30% sucrose. Microelectrodes were removed, and brains were then cryopreserved in optimal cutting temperature compound (OCT). Horizontal tissue sections (16 μm thick) were collected. Immunohistochemistry (IHC) on Layers V and VI was utilized to assess neuronal density in the brain tissue slices. Image analysis was performed according to the Methods section of this paper with background defined as 300–350 μm from the electrode interface.

Reference

1. Kozai, T.D.Y.; Marzullo, T.C.; Hooi, F.; Langhals, N.B.; Majewska, A.K.; Brown, E.B. and Kipke, D.R. Reduction of neurovascular damage resulting from microelectrode insertion into the cerebral cortex using in vivo two-photon mapping. *J. Neural Eng.* **2010**, *7*, 046011.
